# Dimensions of cognition, behaviour, and mental health in struggling learners: A spotlight on girls

**DOI:** 10.1002/jcv2.12082

**Published:** 2022-10-11

**Authors:** Jacalyn Guy, Silvana Mareva, Grace Franckel, the CALM Team, Joni Holmes

**Affiliations:** ^1^ Medical Research Council Cognition & Brain Sciences Unit University of Cambridge Cambridge UK; ^2^ School of Psychology University of East Anglia Norwich UK

**Keywords:** behaviour, cognition, learning difficulties, mental health, sex differences, transdiagnostic

## Abstract

**Background:**

Fewer girls than boys are identified as struggling at school for suspected problems in attention, learning and/or memory. The objectives of this study were to: i) identify dimensions of cognition, behaviour and mental health in a unique transdiagnostic sample of struggling learners; ii) test whether these constructs were equivalent for boys and girls, and; iii) compare their performance across the dimensions.

**Methods:**

805 school‐aged children, identified by practitioners as experiencing problems in cognition and learning, completed cognitive assessments, and parents/carers rated their behaviour and mental health problems.

**Results:**

Three cognitive [Executive, Speed, Phonological], three behavioural [Cognitive Control, Emotion Regulation, Behaviour Regulation], and two mental health [Internalising, Externalising] dimensions distinguished the sample. Dimensions were structurally comparable between boys and girls, but differences in severity were present: girls had greater impairments on performance‐based measures of cognition; boys were rated as having more severe externalising problems.

**Conclusions:**

Gender biases to stereotypically male behaviours are prevalent among practitioners, even when the focus is on identifying cognitive and learning difficulties. This underscores the need to include cognitive and female‐representative criteria in diagnostic systems to identify girls whose difficulties could go easily undetected.

## Graphical abstract

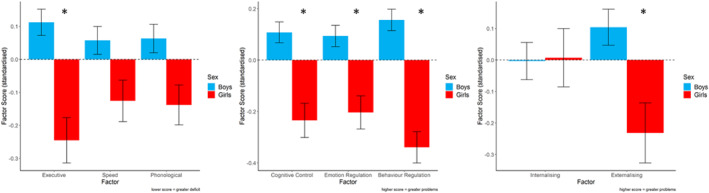

Male and female phenotypes were characterised in a large transdiagnostic sample of children identified by practitioners as experiencing problems in cognition and learning, irrespective of diagnostic status. Dimensions of cognition, behaviour and mental health were structurally invariant between boys and girls, but cognitive problems were more severe in girls, and behavioural difficulties and externalising problems greater in boys.

**Key points**

- Our understanding of sex differences in at‐risk developmental populations is dominated by studies of clinical samples, likely biasing knowledge towards a male phenotype
- Male and female phenotypes were characterised in a large transdiagnostic sample of children identified by practitioners as experiencing problems in cognition and learning, irrespective of diagnostic status
- Practitioners recognised more boys than girls as struggling
- Dimensions of cognition, behaviour and mental health were structurally invariant between boys and girls, but performance‐based cognitive problems were more severe in girls, and behavioural difficulties and externalising problems greater in boys
- These findings illustrate the profile of struggling girls and highlight systemic and implicit biases in the fields of healthcare and education that need to be addressed to provide appropriate support

Approximately 15% of children have special educational needs (SENs), and the majority of these are boys (Department of Education, 2020). Diagnoses of developmental disorders associated with learning problems, including Attention Deficit Hyperactivity Disorder (ADHD) and Autism Spectrum Disorder (ASD), are made earlier and more often for boys. Clinical estimates often indicate a higher boy: girl ratio for ADHD and ASD (e.g., Polanczyk & Rohde, 2007; Maenner et al., 2020). However, there is increasing recognition that these sex^1^ differences may not reflect the number of girls who are struggling, with lower ratios reported in community and population studies (Gershon, 2002; Loomes et al., 2017). These discrepancies likely reflect both the different manifestations of learning and developmental difficulties between sexes, and the use of diagnostic rubrics to identify children's difficulties. The primary aim of the current study is to investigate whether dimensions of cognition, behaviour and mental health differ between boys and girls in a large mixed sample of struggling learners with and without diagnosed difficulties who were identified by health and education practitioners as having problems in attention, learning and/or memory.

## WHY MORE BOYS THAN GIRLS?

One possibility for the high male: female sex ratio might relate to a reliance on diagnostic systems that have emerged from descriptions and categorisations of overt behaviours (discussed in Kreiser & White, 2014; Mowlem, Agnew‐Blais, et al., 2019). Criteria related to overt and externalising behaviours, such as hyperactivity for ADHD, are likely recognised more easily by practitioners and tolerated less well by parents and teachers (Gaub & Carlson, 1997). Add to that socially constructed gender‐biased or stereotypical views of boys as being disruptive, the problem becomes apparent: overt behaviours are more likely to raise concern and expected to be more prevalent in boys. Indeed, studies have shown that boys are rated as more disruptive (e.g., Sciutto et al., 2004).

Another explanation is that symptom manifestations differ between boys and girls. The way in which girls express their difficulties may preclude diagnoses, or make their challenges more difficult to detect. Girls with ADHD often present with predominantly inattentive and internalising symptoms (Biederman et al., 2002; Levy et al., 2005; Rucklidge & Tannock, 2001). In contrast, boys typically present with hyperactive/impulsive symptoms and externalising behaviours (Abikoff et al., 2002; Quinn, 2008). Similarly, some autistic^2^ girls use behavioural camouflaging strategies, appearing less autistic in social interactions (Dean et al., 2017; Hull et al., 2020) and more able from others' perspectives (Hiller et al., 2014), meaning they are less likely to receive a diagnosis. The same is also true in schools, where boys are more likely to manifest externalising behaviours and receive a referral for special education assessment (Dhuey & Lipscomb, 2010).

## SEX DIFFERENCES IN AT‐RISK DEVELOPMENTAL POPULATIONS

Extant research investigating sex differences in cognition, behaviour and mental health in developmental populations has produced mixed results (e.g., Duvall et al., 2020; Gershon, 2002; Gur & Gur, 2017; Gur & Gur, 2016; Mandy et al., 2012; Mayes et al., 2020; Rucklidge, 2008). While some meta‐analyses report that girls show more cognitive impairments than boys (see Gershon, 2002; Gur & Gur, 2016; Gur & Gur, 2017; Hull et al., 2017), other large scale studies report no differences between boys and girls (e.g., Duvall et al., 2020), or alternatively report strengths in language and memory for girls, and spatial tasks and speed for boys (Gur & Gur, 2016, 2017). Similarly, while externalising behaviours such as conduct problems, hyperactivity and aggression are reported to be more common in boys (e.g., Biederman et al., 2002; Mandy et al., 2012; Willcutt & Pennington, 2000), and internalising problems such as anxiety and depression more so in girls (Gershon, 2002; Hull et al., 2017; Kreiser & White, 2014), this is not always the case (e.g., Lecavalier, 2006; Mayes & Calhoun, 2011; Mayes et al., 2020, 2011; Murphy et al., 2009; Rucklidge, 2008). In their review of sex differences in ADHD, for example, Rucklidge (2008) noted that, although aggression and externalising behaviours were generally more common in boys than in girls, these findings were not always consistent across studies. Mayes et al. (2011, 2020) made similar observations for autistic children; boys and girls did not differ in externalising and internalising problems.

One striking and unifying feature is that much of this literature is dominated by the study of clinical samples of diagnosed children (e.g. discussed in Kreiser & White, 2014; Mowlem, Agnew‐Blais, et al., 2019, Mowlem, Rosenqvist, et al., 2019). This likely biases our understanding towards a male phenotype, both because clinical samples typically include a greater number of boys, and because girls included in such samples are likely to present with overt behaviours more similar to those typically expressed in boys (Mowlem, Rosenqvist, et al., 2019). Recruiting selective samples based on the presence of a particular diagnosis means that we do not understand sex differences either in children with milder needs, or in those with more complex and co‐occurring needs who are often excluded from studies on the basis that their comorbid problems are considered a confound.

Developmental problems are increasingly studied using transdiagnostic approaches. These aim to identify dimensions of difficulty that occur across individuals irrespective of diagnostic status (Cuthbert & Insel, 2013). The dimensions studied focus on characteristics and mechanisms that may not align with any conventional diagnostic category (Astle et al., 2021). A spectrum of study designs can yield transdiagnostic insights, including those that recruit via functional definition. These relax recruitment criteria to sample broader populations of individuals with additional needs who would not necessarily be represented in diagnostic‐based approaches: they replace diagnostic criteria‐based selection with sampling based on functionally defined needs (Astle et al., 2021). This approach offers an important alternative way to understand sex differences in struggling learners. Rather than focussing on children with diagnoses informed by diagnostic criteria, which may be biased towards stereotypically male behaviours, transdiagnostic sampling based on functional needs provides the opportunity to recruit children with a broader range of developmental and learning difficulties (e.g., Casey et al., 2014): it provides a way to explore sex differences in the common struggling learner, who may not conform in presentation to standard “male‐biased” diagnostic criteria, as well as children who have existing diagnoses.

## THE CURRENT STUDY

The current study adopts a transdiagnostic, functional needs‐based, approach to characterise male and female phenotypes in a large mixed sample of children described as struggling in school. The goal was to recruit a highly heterogeneous sample of children varying in both the severity and nature of their learning‐related problems, which was not biased towards classically “male” behaviours. This could not be achieved using the recruitment methods usually applied in the sex differences literature: depending exclusively on children with recognized disorders through specialist clinics would exclude children with difficulties that are not captured by diagnostic rubrics, which are likely to be girls who are struggling, but who do not present with overt behavioural problems.

The sample included children who were identified as experiencing problems in attention, learning and/or memory by education and health professionals. It included children with relatively mild problems judged to be compromising their academic progress, who would likely not meet diagnostic thresholds, in addition to many children whose more marked problems would: some children had a single diagnosis, others had multiple diagnoses, but the majority were undiagnosed despite coming to the attention of a professional for experiencing difficulties that were affecting their school progress. By adopting a transdiagnostic perspective, we were therefore able to include children who are not currently represented in the literature on sex differences, specifically those with milder problems who are unlikely to meet diagnostic thresholds, those with presentations that did not fit the “male‐biased” behaviours defined by diagnostic criteria, and those with complex and co‐occurring problems. This enabled us to: i) test whether recruitment based on functional needs rather than diagnostic status replicated the high boy:girl ratio documented in studies using diagnosis‐based recruitment, and ii) explore whether there are sex differences in the types and severity of problems experienced by this broader population of children who are struggling.

Consistent with data‐driven approaches adopted across transdiagnostic studies (e.g., Holmes et al., 2021; Kotov et al., 2017; Mercier et al., 2018; Reininghaus et al., 2019; Sokolova et al., 2017) a latent variable approach was used to identify dimensions of difficulty in the whole sample, and then to test whether these dimensions differed in structure and severity between boys and girls. Identifying dimensions side‐steps debates about which of two different measures sharing common variance represents a core deficit or difference, and instead identifies the major sources of variance across all measures in a dataset. In this case, the broad dimensions of cognition, mental health and behaviour that may or may not differ between boys and girls.

We broadly classified multiple individual tasks and behaviour ratings into three domains: cognition, behaviour, and mental health. Performance‐based tasks capturing the processing efficiency of cognitive abilities in structured conditions were used to index function in the cognitive domain. The tasks selected for inclusion were those measures that were administered to the whole sample, and which were included in a study that previously identified the cognitive dimensions differentiating performance in this sample (see Holmes et al., 2020). This earlier study identified three cognitive dimensions, executive function, processing speed and phonological processing, using age‐normed scores (Holmes et al., 2020). Here we included the same tasks and participants but use age‐regressed raw scores in place of the age‐normed scores used by Holmes et al. (2020) because some of the measures factor sex into their age standardization (Mayes et al., 2020). In the interests of replication, we adopted the same analytic approach as Holmes et al. (2020) to identify cognitive dimensions using these scores.

Despite some content overlap, parent ratings of behaviour were categorised a priori as either behaviour or mental health (see Table S1), based on both common uses of the measures in research (Alloway et al., 2009; Fink et al., 2015; Patalay et al., 2015) and their use in clinical and educational practice. Scales used widely to measure externalising and internalising symptoms were classified as mental health, while those capturing symptoms associated with cognitive or neurodevelopmental difficulties were classified as behaviour. The parent ratings of behaviour included observations of the children's cognitive behaviours in everyday settings. We use the terminology of our test instruments to describe the observed measures of cognitive function throughout, meaning the same terms (e.g., executive functions or working memory) are used to refer to both objectively measured cognitive abilities that we have classed as “cognitive” and to subjective ratings of cognitive behaviours that we have classed as “behaviour”. Despite this overlap in terminology, we conceptualise objective cognitive task performance and everyday cognitive behaviours as separate constructs, consistent with an extensive literature suggesting they provide non‐overlapping information, and that functioning in the two domains makes independent contributions to clinical and academic problems (Soto et al., 2020; Toplak et al., 2013).

Existing evidence for differences in the manifestation of cognitive, behavioural and mental health problems between boys and girls with neurodevelopmental problems is mixed. This, combined with the unique nature of our sample, motivated our choice to conduct all analyses in a data‐driven and exploratory fashion. We, therefore, had no specific predictions about whether the factor compositions would be similar for male and female struggling learners, or whether the severity of impairments would differ between boys and girls on specific dimensions.

## METHOD

### Procedure and measures

The cognitive, behavioural and mental health data from the Centre for Attention, Learning and Memory (CALM) cohort were used (see Table S1 for a description of the tasks). Recruitment details and testing procedures are described in the study protocol (Holmes et al., 2019). Ethical approval was granted by the National Health Service (REC: 13/EE/0157). Parents/caregivers provided written consent and child verbal assent was obtained.

Raw scores were used in all analyses as some measures factor sex into their age standardization (Mayes et al., 2020). To control for age, raw scores were regressed on age and the residuals were used. Higher raw scores were associated with better performance for the cognitive tasks, but greater severity for the behavioural and mental health questionnaires. Residuals for Rapid Naming, Simple Reaction Time (SRT; TEA‐Ch2; Manly et al., 2016) and Prosocial Behaviour (SDQ; Goodman, 1997) were reverse coded to streamline the interpretation of respective cognitive and mental health measures. Missing data were imputed with a full information maximum likelihood estimator for all models (Rosseel, 2012).

### Participants

The sample included 805 children, whose average age was *M* = 9.48 years, *SD* = 2.38 (552 boys *M*_age_ = 9.41, *SD* = 2.35; 253 girls *M*_age_ = 9.63, *SD* = 2.46, *t*(468.6) = −1.21, *p* = 0.23). Characteristics, including diagnostic status and referral route, are presented in Table S2.

Performance was close to age‐appropriate levels for Mr X and Cancellation (see Table 1). All other cognitive scores were approximately one standard deviation below age‐normed population means. Behavioural problems were elevated for the whole sample (see Table S3), with the exception of Organisational problems (BRIEF; Gioia et al., 2000). The majority of mental health ratings on the RCADS‐P (Chorpita et al., 2000) were elevated, but still within age‐typical and subclinical bounds (i.e. RCADS‐P T score less than 65).

**TABLE 1:** Descriptive statistics for boys and girls on cognitive, behavioural and mental health measures with residual scores

	Group												
	Boys					Girls					Group comparisons		
Measures	*N*	*M*	*SD*	Min	Max	*N*	*M*	*SD*	Min	Max	*t*	*p*	*d*
**Cognition**													
Alliteration	539	0.07	2.26	−9.24	3.94	249	−0.16	2.17	−6.81	3.44	1.40	0.16	−0.11
Rapid naming	538	2.36	33.14	−163.69	83.33	248	−5.13	37.71	−221.02	50.59	2.69	0.01*	−0.22
Nonword repetition	339	0.20	6.22	−18.21	12.78	142	−0.47	5.82	−15.46	11.79	1.13	0.26	−0.11
Digit recall	550	0.19	4.92	−16.70	17.93	251	−0.41	4.95	−18.99	15.77	1.59	0.11	−0.12
Dot matrix	547	0.35	4.89	−19.95	20.81	252	−0.76	4.41	−15.28	11.39	3.18	<0.001***	−0.23
Backward digit recall	534	0.21	3.62	−11.09	14.56	246	−0.45	3.62	−12.18	10.16	2.33	0.02*	−0.18
MrX	544	0.55	4.35	−13.54	18.23	251	−1.19	4.28	−13.38	13.71	5.31	<0.001***	−0.40
Following instructions	516	0.07	3.84	−9.60	18.58	234	−0.16	3.16	−9.11	9.10	0.87	0.38	−0.06
Delayed recall	530	0.48	14.82	−39.08	36.50	245	−1.03	15.25	−47.49	34.58	1.30	0.20	−0.10
Cancellation	525	0.10	3.28	−9.89	10.12	246	−0.21	3.47	−10.10	9.04	1.16	0.25	−0.09
SRT	501	0.19	274.20	−1373.14	465.06	238	−0.40	253.58	−1047.39	451.09	0.03	0.98	0
Vigil/Barking	514	0.01	2.37	−7.01	3.35	234	−0.03	2.45	−7.32	3.35	0.22	0.82	−0.02
Matrix reasoning	551	0.29	4.58	−14.24	13.00	253	−0.64	4.46	−12.24	14.56	2.73	0.01*	−0.21
**Behaviour**													
BRIEF:Inhibit	548	0.77	6.01	−12.14	9.37	252	−1.68	6.11	−11.98	9.88	5.30	<0.001***	−0.41
BRIEF:Shift	549	0.35	4.17	−9.22	7.32	252	−0.76	4.27	−9.27	7.37	3.43	<0.001***	−0.26
BRIEF:Emotion control	548	0.37	5.44	−12.23	7.91	252	−0.80	5.76	−12.13	7.90	2.70	0.01*	−0.21
BRIEF:Initiate	548	0.16	3.14	−10.29	6.74	252	−0.36	3.51	−8.92	6.36	2.02	0.04*	−0.16
BRIEF:WM	548	0.19	3.93	−14.72	4.51	252	−0.42	4.26	−14.63	4.65	1.94	0.05	−0.15
BRIEF:Planning	539	0.55	4.80	−16.14	7.91	250	−1.19	5.07	−14.71	8.23	4.55	<0.001***	−0.36
BRIEF:Organisation	549	0.02	3.22	−9.01	3.63	252	−0.04	3.25	−8.99	3.62	0.22	0.83	−0.02
BRIEF:Monitor	547	0.37	3.37	−10.08	5.40	252	−0.81	3.69	−9.95	5.39	4.33	<0.001***	−0.34
CPSF:Inattention	544	0.31	3.20	−11.66	3.51	251	−0.67	3.83	−11.72	3.56	3.50	<0.001***	−0.29
CPSF:Hyperactivity/Impulsivity	546	0.79	5.28	−11.78	8.71	251	−1.73	5.57	−11.68	10.58	6.03	<0.001***	−0.47
CPSF:Executive function	544	0.37	3.50	−10.64	5.27	250	−0.80	3.70	−11.00	5.27	4.17	<0.001***	−0.33
CPSF: Aggression	544	0.29	4.04	−3.78	11.52	250	−0.63	3.80	−3.71	11.36	3.10	<0.001***	−0.23
CPSF:Peer relations	539	0.20	4.37	−7.73	9.92	249	−0.43	4.50	−7.04	10.51	1.83	0.07	−0.14
**Mental health**													
RCADS:Generalised anxiety	274	0.04	4.04	−6.34	12.18	124	−0.09	3.81	−6.55	10.11	0.29	0.77	−0.03
RCADS:Panic disorder	273	−0.11	3.99	−4.83	22.24	123	0.25	4.30	−4.95	18.86	−0.78	0.43	0.09
RCADS:Social phobia	276	−0.32	6.19	−14.13	14.62	124	0.71	6.39	−12.11	14.42	−1.51	0.13	0.17
RCADS:Separation anxiety	277	0.02	4.85	−7.99	13.41	125	−0.05	5.31	−7.62	13.04	0.14	0.89	−0.02
RCADS:Obsessive‐compulsive	275	0.06	3.10	−3.02	11.99	124	−0.14	3.05	−3.02	11.99	0.62	0.54	−0.07
RCADS:Major depression	276	0.16	5.17	−9.89	14.97	125	−0.34	4.89	−9.04	17.38	0.93	0.35	−0.10
SDQ:Conduct problems	546	0.19	2.51	−3.68	6.67	251	−0.41	2.54	−3.85	6.60	3.10	<0.001***	−0.24
SDQ:Prosocial behaviour	546	0.26	2.31	−3.37	6.98	251	−0.56	2.41	−3.60	5.98	4.52	<0.001***	−0.35
SDQ:Peer relationships problem	546	0.09	2.55	−5.20	6.97	251	−0.19	2.63	−4.65	6.82	1.37	0.17	−0.11
SDQ:Hyperactivity/Inattention	545	0.32	2.30	−7.59	3.12	251	−0.69	2.52	−7.84	2.93	5.37	<0.001***	−0.42

*Note*: Descriptive statistics are based on age regressed residuals on raw scores. All performance‐based assessments were classified as cognitive, and all subjective rating‐based assessments were classified as behaviour or mental health based on common use in the literature. For the cognitive tasks, lower scores indicate greater difficulties. For all behavioural and mental health tasks, higher ratings reflect greater difficulties. Residuals for Rapid Naming, Simple Reaction Time (SRT; TEA‐Ch2) and Prosocial Behaviour (SDQ) were reverse coded to streamline the interpretation of respective cognitive and mental health measures.

Abbreviations: BRIEF, The Behaviour Rating Inventory of Executive Function; CPSF, Conners‐3 Parent Rating Scale Short Form; RCADS, Revised Child and Anxiety and Depression Scale (Parent Version); SDQ, Strengths and Difficulties Questionnaire.

*p < 0.05, ***p < 0.001.

### Analysis plan

Analyses were conducted in four steps: exploratory factor analysis (EFA), confirmatory factor analysis (CFA), multigroup CFA with measurement invariance (Steenkamp & Baumgartner, 1998; van de Schoot et al., 2013), and comparisons of latent means (intercepts). A detailed description of this approach is provided in the Supporting Information. Parallel analysis was used to determine the maximum number of factors to extract in EFAs for the cognitive, behavioural and mental health data. Parallel analysis involves simulations that create random datasets with properties similar to the true data: estimated numbers of factors are extracted and compared to a permuted baseline, and extraction is stopped when eigenvalues fall within the 95% confidence interval of eigenvalues from the simulated data, revealing the optimal number of factors to extract from the true data. For the EFA, factor structures were considered interpretable if they provided a good fit to the data and there was a minimum of two primary loadings per latent construct (note that two loadings are considered acceptable with large sample sizes, Costello & Osborne, 2005). The labelling of the factors reflected the constellation of the highest loading variables. For the cognitive domain (performance‐based tasks), the factor structure and labelling was based on a previous study using the same cohort data and cognitive tasks (see Holmes et al., 2020). All analyses were conducted using R version 4.0.3 using the Psych (2.0.12; Revelle, 2020), Lavaan (0.6–7; Rosseel, 2012) and semTools packages (0.5–4, Jorgensen et al., 2020).

## RESULTS

### Descriptive statistics

Descriptive statistics for boys and girls are presented in Table 1. Additional descriptive statistics for the whole sample are provided in Table S3. Correlations between the measures are provided in Table S4–S6.

Considerably more boys than girls were referred: 552 boys and 253 girls. Comparisons between boys and girls revealed girls performed more poorly than boys on the majority of cognitive measures (see Table 1). Boys were rated higher than girls on most of the behaviour rating scales, except WM (BRIEF; *p* = 0.05), Organisation (Conners Parent Short Form (CPSF); Conners, 2008; *p* = 0.83), and Peer Relations (CPSF; *p* = 0.07; see Table 1). Boys also had elevated ratings on mental health subscales measuring conduct problems, hyperactivity, and prosocial behaviours (all SDQ; all *ps* < 0.05; see Table 1).

### Dimensions of cognition, behaviour and mental health

#### Cognition

Parallel analysis followed by an EFA with an oblimin rotation suggested a four‐factor model was a good fit to the cognitive data, χ2 (32) = 63.47, *p* < 0.001, RMSEA = 0.035 (90% confidence interval [CI] = 0.022, 0.048), CFI = 0.986, RMSR = 0.02; see Schermelleh‐Engel et al., 2003). This model included factors corresponding to executive function (Matrix Reasoning, Following Instructions, Mr X), verbal short‐term (STM) and working memory (WM, Digit Recall, Nonword Repetition, Backward Digit Recall), phonological processing and attention (Alliteration, Rapid Naming, Cancellation, SRT, Vigil), and visual STM (Dot Matrix; see Table S7). Despite the good fit, this model was difficult to interpret because the fourth factor representing visual STM had only one indicator and the factor reflecting phonological processing and attention had loadings from measures with little in common. For these reasons, a three‐factor model was tested.

Fit statistics for a three‐factor solution indicated that this model was a good fit, χ2 (42) = 105.5, *p* < 0.001, RMSEA = 0.044 (90% confidence interval [CI] = 0.033, 0.054), CFI = 0.947, RMSR = 0.030, see Table S7). The first factor was most strongly associated with measures that draw on executive resources (Dot Matrix, Backward Digit Recall, Mr X and Matrix Reasoning). The second factor was linked mostly to speeded tasks or tasks that were completed under time constraints (SRT, Rapid Naming, Cancellation, and Vigil). This factor was also linked to tasks that were not speeded (Alliteration, Delayed Recall, and Following Instructions), but that might be performed better if they are performed quickly (e.g., due to less forgetting time). The third factor was associated with measures involving the storage of phonological material (Digit Recall and Nonword Repetition). These factors were labelled Executive, Speed, and Phonological Processing, respectively. These labels are indicative and consistent with those used in Holmes et al. (2020) who conducted a similar analysis on the same variables from the same cohort using age‐standardised scores. The labelling is based on a cognitive analysis of the highest‐loading variables on each latent construct. As such the terms used broadly capture the cognitive composition of the factors, but some of the variable loadings do not align fully with this reductive nomenclature. For example, the labelling of Factor 2 as Speed does not necessarily reflect that all tasks were speeded, but instead that the majority of tasks with a speeded component loaded on this factor. A CFA revealed the three‐factor model was an acceptable fit to the data, χ2(62) = 203.12 *p* < 0.001, RMSEA = 0.053 (90% confidence interval [CI] = 0.045, 0.061), CFI = 0.930, SRMR = 0.039, so it was selected for further analyses exploring sex differences.

#### Behaviour

Parallel and EFA analyses identified a three‐factor solution as an acceptable fit to the behavioural data (see Table S8), χ2(42) = 417.75, *p* < 0.001, RMSEA = 0.105 (90% confidence interval [CI] = 0.096, 0.115), CFI = 0.947, RMSR = 0.03). The Monitor subscale loaded on all three factors in this model, and excluding it yielded a marginally better fit, χ2 (33) = 311.50, *p* < 0.001, RMSEA = 0.102 (90% confidence interval [CI] = 0.092, 0.113), CFI = 0.956, RMSR = 0.03). This latter model, excluding the Monitor subscale, was selected for further analysis in the interest of parsimony, Δχ2(9) = 106.24, *p* < 0.001. Symptoms related to everyday difficulties with cognitive control loaded most highly on Factor 1. These included WM, planning, executive functions, inattention, initiation, and organization. Problems with emotional control, shifting, aggression, and peer relationships loaded most highly on Factor 2. Subscales measuring behavioural control, including hyperactivity and inhibition, loaded on Factor 3. The factors were labelled Cognitive Control (Factor 1), Emotion Regulation (Factor 2) and Behaviour Regulation (Factor 3). These labels were given based on the hypothesized dimension underlying differences in performance based on the constellation of tasks with the highest loadings. It is important to note that they do not represent a rigid mapping of tasks on factors and could be labelled differently depending on the sample (e.g., Simpson‐Kent et al., 2020).

A CFA testing the fit of the three‐factor model indicated it was a poor fit to the data, χ2(51) = 676.21, *p* < 0.001, RMSEA = 0.124 (90% confidence interval [CI] = 0.115, 0.132), CFI = 0.900, SRMR = 0.054. Modification indices revealed that scores for the Initiation subscale loaded highly on two factors: Emotion Regulation and Cognitive Control. Allowing this measure to cross‐load on both factors significantly improved the goodness of fit, Δχ2(1) = 52.43, *p* < 0.001, but the overall model remained an inadequate fit, χ2(50) = 615.08, *p* < 0.001, RMSEA = 0.119 (90% confidence interval [CI] = 0.110, 0.127), SRMR = 0.047, CFI = 0.909. Further inspection of the modification indices suggested that allowing hyperactivity and inattention, symptoms that co‐occur in ADHD, to co‐vary might improve the model. Adding this covariance significantly improved the model, Δχ2(1) = 113.36, *p* < 0.001, and produced a model with adequate fit to use in subsequent analyses exploring sex differences, χ2(49) = 488.98, *p* < 0.001, RMSEA = 0.106 (90% confidence interval [CI] = 0.097, 0.114), SRMR = 0.043, CFI = 0.929.

#### Mental health

A parallel analysis indicated a two‐factor solution would be a good fit, which was confirmed by an EFA, χ2(26) = 59.4, *p* < 0.001, RMSEA = 0.056 (90% confidence interval [CI] = 0.038, 0.076), CFI = 0.981, RMSR = 0.02; see Table S9). Factor 1 was associated with symptoms of generalised, social and separation anxiety, panic and obsessive‐compulsive disorders, and depression. Factor 2 was linked to conduct and peer relationship problems, low prosocial behaviour, and hyperactivity. These factors were labelled Internalising (Factor 1) and Externalising (Factor 2), respectively. CFA fit indices revealed that this two‐factor model was an acceptable fit to the data, χ2(34) = 179.44, *p* < 0.001, RMSEA = 0.103 (90% confidence interval [CI] = 0.088, 0.118), SRMR = 0.069, CFI = 0.918, with the exception of the RMSEA statistic (e.g. > 0.08). Modification indices suggested that allowing depression to cross‐load on both factors would improve the model. The model was re‐estimated with this cross‐loading, and the fit was improved significantly, Δχ2(1) = 75.49, *p* < 0.001, yielding fit statistics within an acceptable range, χ2(33) = 111.72, *p* < 0.001, RMSEA = 0.077 (90% confidence interval [CI] = 0.062, 0.093), SRMR = 0.054, CFI = 0.956. This model was used in all subsequent analyses.

### Comparing boys and girls

#### Cognition

Tests of configural invariance revealed that the three‐factor model captured the data well for both boys and girls, χ2(124) = 280.70, *p* < 0.001, RMSEA = 0.056 (90% confidence interval [CI] = 0.047, 0.065), SRMR = 0.046, CFI = 0.922. Tests of metric invariance showed no significant deterioration of model fit when loadings were constrained to be equal across groups, Δχ2(10) = 11.48, *p* = 0.321. However, tests of scalar invariance indicated that the intercepts differed between boys and girls, Δχ2(10) = 18.86, *p* = 0.04. Inspection of the modification indices revealed a large discrepancy for the MrX test. Releasing the equality constraint on these intercepts improved the model fit to the data, Δχ2(9) = 7.85, *p* = 0.550, and partial scalar invariance was supported (see Table S10).

**FIGURE 1:**
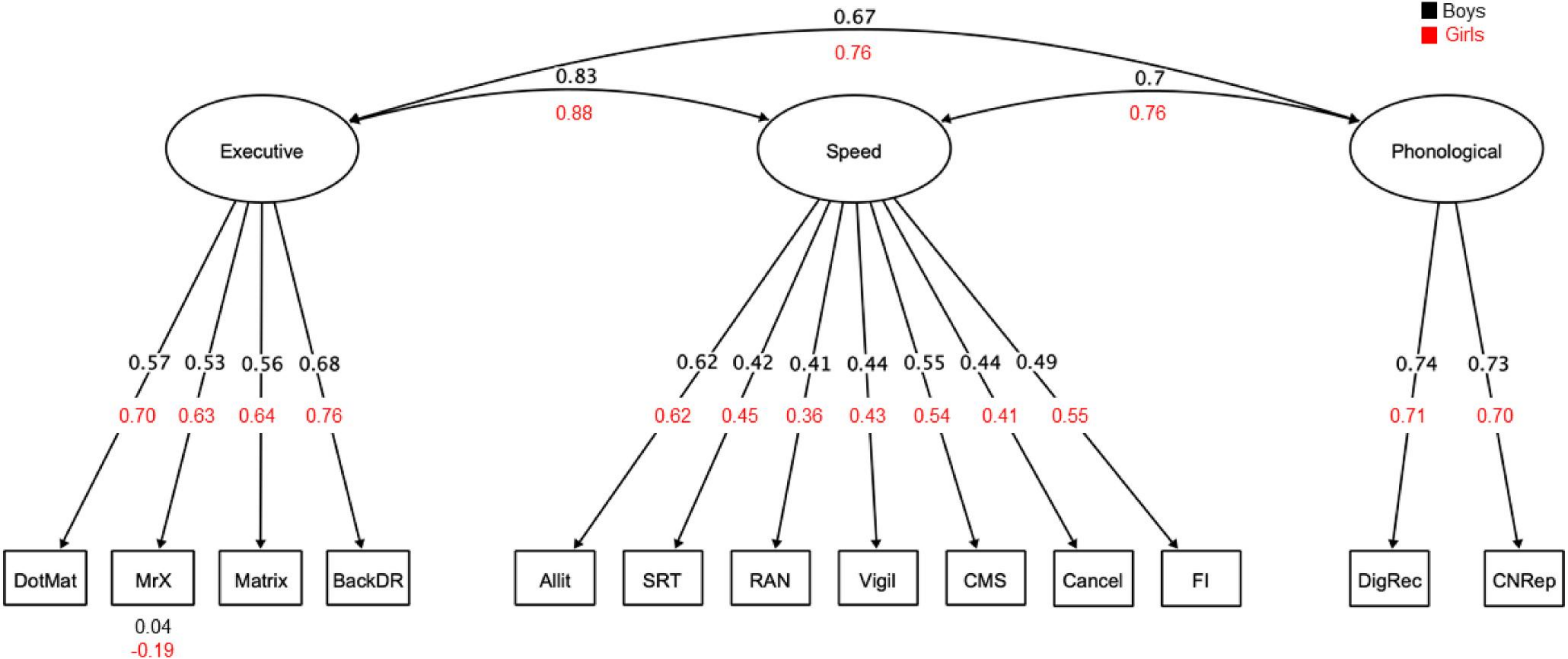
Multigroup measurement model of cognitive dimensions. The cognitive measurement model included only performance‐based assessments. Standardized values are derived from the model that achieved partial scalar invariance across boys and girls. For ease of interpretation, only intercepts that differed significantly across groups are shown. These intercepts were estimated freely across groups. Allit, Alliteration; BackDR, Backward Digit Recall; Cancel, Cancellation; CMS, Delayed Recall; CNRep, Nonword Repetition; DigRec, Digit Recall; DotMat, Dot Matrix; Executive, Executive Functions; FI, Following Instructions; Matrix, Matrix Reasoning; MrX, Mister X; Phonological, Phonological Processing; RAN, Rapid Automatic Naming; Speed, Processing Speed; SRT, Simple Reaction Time

Group differences in latent means were explored by comparing the freely estimated and constrained models (see Figure 1). The freely estimated model fit better than the constrained model, suggesting that latent intercepts (i.e. means) differed between boys and girls. Applying individual constraints on the latent intercepts revealed a significant difference between groups on the Executive factor alone, Δχ2(1) = 11.62, *p* < 0.001, with girls performing significantly worse than boys (girls *M* = −0.49, SD = 2.87, boys *M* = 0.44, SD = 2.44). Intercepts for Speed and Phonological Processing did not differ significantly between groups (Speed Δχ2(1) = 3.46, *p* = 0.06, Phonological Processing Δχ2(1) = 3.48, *p* = 0.06; see Figure 4).

#### Behaviour

**FIGURE 2:**
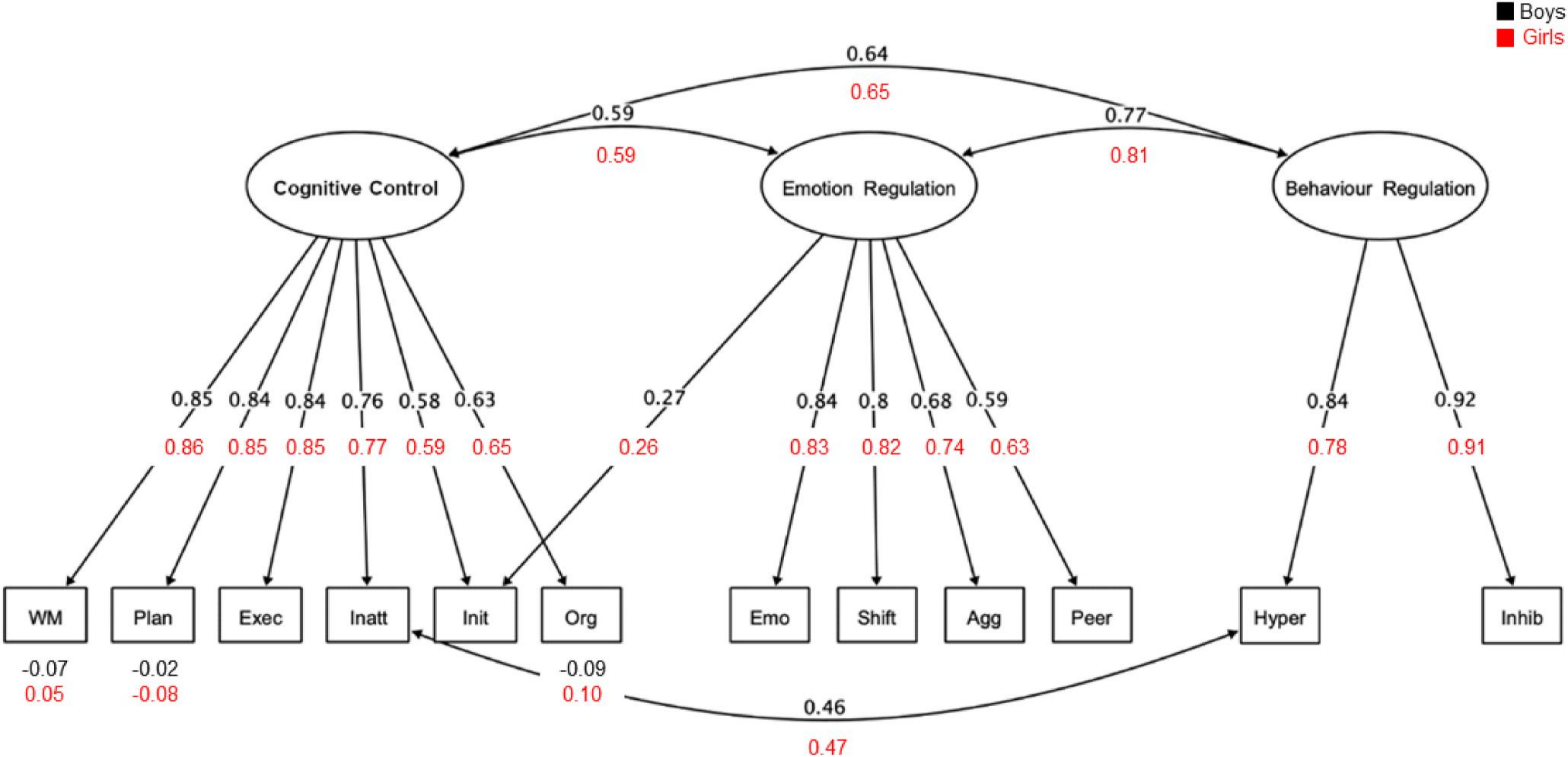
Multigroup measurement model of behaviour dimensions. The behaviour measurement model was exclusively based on subjective parental ratings. Standardized values are derived from the model that achieved partial scalar invariance across boys and girls. For ease of interpretation, only intercepts that differed significantly across groups are shown. These intercepts were estimated freely across groups. Agg, Aggression; Emo, Emotion Control; Exec, Executive Functions; Executive, Executive Functions; Hyper, Hyperactivity; Inatt, Inattention; Inhibit, Inhibition; Org, Organisation; Peer, Peer Relations; Plan, Planning; Shift, Shifting; WM, Working Memory

The behavioural model met conditions for configural and metric invariance but not for scalar invariance (see Table S10). Modification indices for subtest mean scores revealed discrepancies for Organisation, Planning and WM. Allowing these intercepts to vary freely between groups improved the model fit and partial scalar invariance was achieved, Δχ2(6) = 11.69, *p* = 0.07 (see Figure 2).

The freely estimated model fit better than the constrained model, indicating that latent intercepts were significantly different between boys and girls. Boys had significantly more difficulties on all three factors, Cognitive Control (Δχ2(1) = 17.56, *p* < 0.001, boys *M* = 0.11, SD = 0.95, girls *M* = −0.24, SD = 1.06), Emotion Regulation (Δχ2(1) = 10.39, *p* = 0.001, boys *M* = 0.09, SD = 0.97, girls *M* = −0.20, SD = 1.03), and Behaviour Regulation (Δχ2(1) = 37.34, *p* < 0.001, boys *M* = 0.16, SD = 0.98; girls *M* = −0.34, SD = 0.97; see Figure 4).

#### Mental health

**FIGURE 3:**
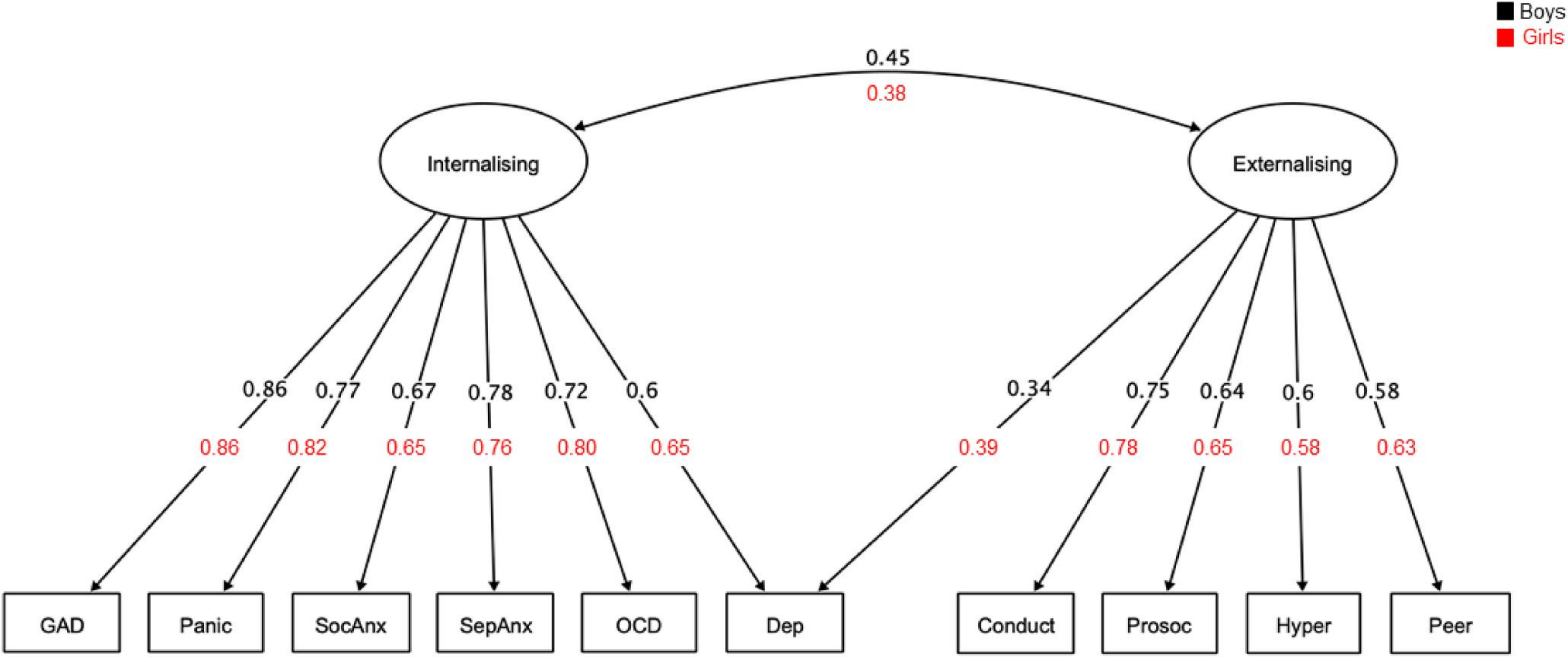
Multigroup measurement model of mental health dimensions. The mental health measurement model was exclusively based on subjective parental ratings. Standardized values are derived from the model that achieved scalar invariance across boys and girls. For ease of interpretation, intercepts are not shown. Conduct, Conduct Problems; Dep, Depression; GAD, Generalized Anxiety Disorder; Hyper, Hyperactivity/Inattention; OCD, Obsessive Compulsive Disorder; Panic, Panic Disorder; Peer, Peer Relationship Problems; ProSoc, Prosocial Behaviour; SepAnx, Separation Anxiety; SocAnx, Social Anxiety

For mental health, there was no significant deterioration of model fit with increasing constraints. The conditions of configural, metric and scalar invariance were met indicating that the overall structure, loadings and intercepts were similar across groups (see Table S10 and Figure 3).

**FIGURE 4:**
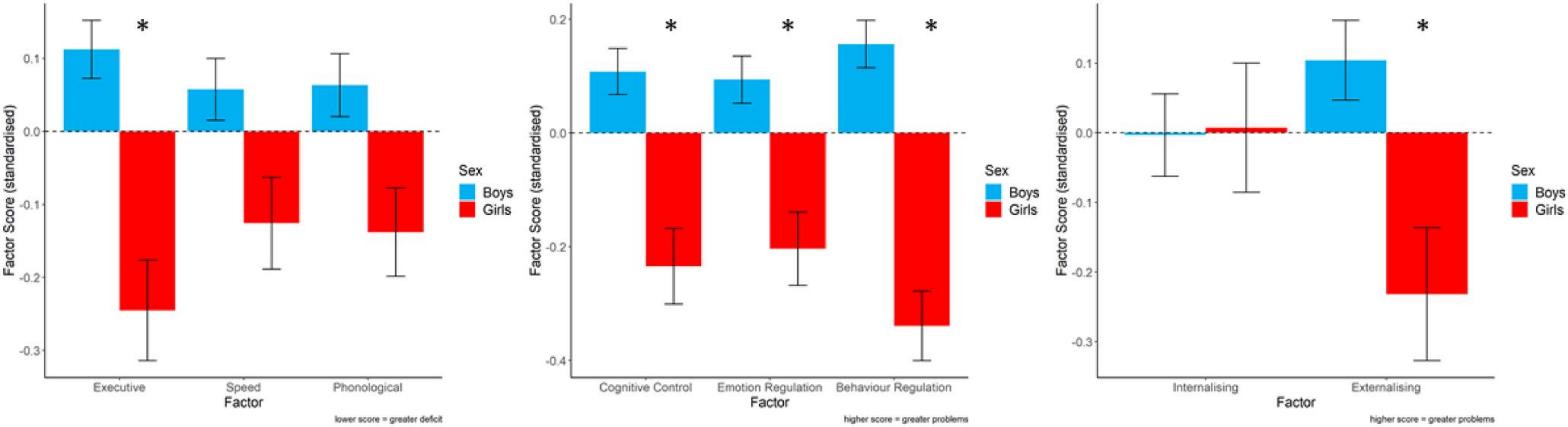
Mean cognitive, behavioural and mental health factor scores for boys and girls. Factor scores were converted to Z scores for visualization. Error bars represent the standard error of the mean. Executive, Executive Functions; Phonological, Phonological Processing; Speed, Processing Speed

Comparisons between boys and girls revealed that constraining intercepts for the Externalising factor significantly degraded the fit, Δχ2(1) = 18.56, *p* < 0.001. Boys exhibited significantly more externalising problems than girls (boys *M* = 0.10, SD = 0.95; girls *M* = −0.23, SD = 1.07). No significant group differences were found for internalising problems, Δχ2(1) = 0.03, *p* = 0.871 (see Figure 4).

## DISCUSSION

This is the first study, to our knowledge, to adopt a transdiagnostic dimensional approach to understanding sex differences in children with developmental difficulties. The key findings were that more boys than girls were referred, and while dimensions of cognition, behaviour and mental health were invariant across boys and girls, cognitive problems were more severe in girls and behavioural difficulties and externalising problems greater in boys.

### Prevalence of boys and girls

Prevalence estimates indicating a high boy:girl ratio for developmental difficulties are drawn predominantly from studies of clinical populations (e.g. autistic children or those with ADHD, discussed in Kreiser & White, 2014; Mowlem, Agnew‐Blais, et al., 2019, Mowlem, Rosenqvist, et al., 2019). Using a novel transdiagnostic sampling frame, which was based on functional need and aimed to represent the full spectrum of children with learning‐related difficulties, including those with problems that are less likely to fit with the behaviours described by diagnostic criteria, we also found a high boy:girl ratio, with twice as many boys than girls referred. This might reflect implicit gender biases and stereotyping (e.g., discussed in Anderson, 1997), or different manifestations, drivers, and expressions of difficulties in boys and girls (e.g., Dhuey & Lipscomb, 2010; Hiller et al., 2014), but we suspect it is also related to practitioners using heuristics for diagnostic criteria that emphasize overt behaviours.

Referrers to this study identified children based on observations of cognitive and learning problems. Despite this, health and education practitioners referred more children with behavioural difficulties than cognitive problems, suggesting they were spotting and raising concern for overt behaviours more easily than cognitive problems. This bias towards male‐focussed diagnostic criteria makes it less likely for a girl to be diagnosed with conditions such as ASD (Lai et al., 2015), and here we see it extends to the broader population of children struggling at school. Moving forward it will be important to decrease these biases towards diagnostic criteria, and increase knowledge of the female phenotype among professionals involved in referrals, to ensure we meet the needs of girls who are struggling.

### Differences between boys and girls

Three broad dimensions underpinning performance on cognitive tasks were associated with measures that were largely spatial or executive (labelled Executive Function), speed‐dependent (labelled Processing Speed) or verbal in nature (labelled Phonological Processing). These factors correspond to those previously identified in a study investigating the cognitive dimensions of learning in the same children from the CALM cohort (Holmes et al., 2020), which used age‐normed scores that factor sex into their standardisation. Using age‐regressed raw scores so as not to mask sex differences, this study shows again that the key constructs that distinguish cognitive abilities in typically developing children and adults also differentiate cognitive test performance in struggling learners.

The cognitive dimensions were invariant across boys and girls, indicating that the overall latent structure of cognitive skills does not differ between sexes. There were no differences in scores on the phonological or speed dimensions, but girls were more impaired on the executive function dimension. These data support the notion that girls must show greater cognitive deficits for educational or health practitioners to notice their struggles (e.g., Dworzynski et al., 2012; Gaub & Carlson, 1997). They also suggest the biggest driver of problems for girls in our sample is performance‐based executive function difficulties. Executive functions are associated with learning outcomes in typical and neurodiverse groups (e.g., Peng et al., 2018; Swanson & Ashbaker, 2000). However, there is evidence that girls may draw more on these resources than boys: girls take a more effortful, mastery‐based approach to learning that draws on general higher‐order cognitive skills, while boys draw more on domain‐specific knowledge and skills during learning (Brunner et al., 2008; Kenney‐Benson et al., 2006). If this is the case, then impairments in executive function problems might be expected to have a more significant impact on girls' school progress, and this might explain why the girls referred to the CALM cohort were characterised by more severe executive function problems than the boys.

This difference was only observed with the performance‐based measures of cognitive abilities, and not with the behaviour rating‐based assessments of cognition. The disconnect between girls' cognitive performance and their behaviour might explain why classroom struggles are often overlooked in girls, and over‐attributed to boys. This may be because girls are either able to mask their cognitive difficulties in their behaviour, or due to the ratings capturing gender biases towards overt behaviours. Speaking to this issue, the referral numbers for both sexes (Table S2) were more similar for education practitioners (66% boys and 34% girls) and speech and language therapists (62% boys and 38% girls) but less similar for referrals via health professionals (75% boys and 25% girls) who are likely better versed in diagnostic criteria that capture more of these overt behaviours.

Parent/carer ratings of the children's behaviour and mental health captured three dimensions of behaviour and two of mental health. For behaviour, factors corresponding to Cognitive Control, Emotion Regulation and Behaviour Regulation emerged. Two of these factors, Behaviour Regulation and Cognitive Control, map on to dimensions of impairment proposed by multiple pathway models of ADHD (e.g., Castellanos et al., 2006). According to these models, ADHD symptoms arise as a consequence of impairments in two neurobiological pathways: one serves cool cognitive functions such as working memory, planning and switching, and the other hot executive functions that contribute to hyperactivity/impulsivity and emotional‐reward dysregulation (Zelazo & Müller, 2002). The third dimension, Emotional Regulation, resembles part of a broader self‐regulation concept, which is also linked to increased risks for ADHD (Walcott & Landau, 2004) and other psychopathologies (e.g., McLaughlin et al., 2011; Röll et al., 2012). For mental health, two dimensions emerged, internalising and externalising. These align with models of child psychopathology (Achenbach, 1966; McElroy, et al., 2018; Patalay et al., 2015).

Dimensions of behaviour and mental health were the same for boys and girls, but the severity of their impairments differed on specific dimensions. There were no sex differences on the internalising symptoms dimension, consistent both with other recent findings from the same cohort (Holmes et al., 2021) and with evidence from other developmental populations (e.g., Mayes et al., 2020). Symptoms on this dimension were elevated for both boys and girls. Internalising problems have been linked to stressful and negative life events (Kim et al., 2003; March‐Llanes et al., 2017), which are likely common among our sample, and may explain why symptoms were elevated for both sexes. These elevated levels may explain why there were no sex differences. Overall, girls had fewer externalising problems and fewer difficulties across all three dimensions of behaviour than boys. This could mean that externalising symptoms and overt behaviours commonly associated with ADHD are genuinely more prevalent and manifest in boys (e.g., Abikoff et al., 2002). Alternatively, elevated problem behaviours in boys could reflect socially constructed gender‐biased or stereotypical views of boys as being disruptive, and the application and use of diagnostic criteria that emphasise overt behaviours (Hiller et al., 2014; Mowlem, Agnew‐Blais, et al., 2019).

### Limitations

While there are many strengths to this study, several limitations need to be acknowledged. Our novel sampling approach broadens the study of sex differences in neurodevelopmental populations to include a more representative sample than is typical, but there are some drawbacks. First, our recruitment approach relied on practitioner referral, opening the possibility of gender bias in referrals. Second, while critical to addressing the study goals, it is unclear whether our findings will generalize to samples recruited using different selection criteria. In terms of assessments, we made a priori choices about the classification of measures as cognitive, behavioural, or mental health considering differences in measurement type (objective task performance or subjective questionnaire rating) and their categorisation and use in both previous studies and in practice. It is possible that classifying our measures in a different way would produce different results, although the sex differences observed at the individual task‐level align with the patterns of differences observed at the dimensional level providing confidence in the primary outcomes. A final issue concerns the labelling of latent factors. For simplicity and clarity, labels were assigned to each factor, as is standard practice in the field. The labels reflected the hypothesized dimension underlying differences in performance, based on the constellation of tasks or subscale scores with the highest loadings on each factor, but they do not reflect a rigid mapping between each measure and each factor. For example, the second factor in the cognitive model is labelled processing speed because the tasks loading most highly on this factor were either speeded tasks (scores were based on RTs) or completed under time constraints. Labelling this factor as processing speed does not imply that either the Alliteration or Following Instructions tasks are measures of processing speed. The challenge of assigning appropriate labels to latent constructs is not unique to this study and does not detract from the benefits of having theory‐guided labels to aid the interpretability of our findings.

## CONCLUSION

This study shows that when health and education professionals identify children with cognitive and learning problems, they recognise more boys than girls. Despite this, girls who were referred showed greater difficulties on performance‐based measures than boys, with significantly greater impairments in executive functioning. They exhibited fewer externalising problems and were rated as having fewer behavioural cognitive difficulties than boys. These results underscore the need to include cognitive and female‐representative criteria in diagnostic systems. Including these criteria, and/or routinely administering performance‐based cognitive assessments in schools may help to identify girls whose difficulties could easily go undetected. By raising awareness of the profile of struggling girls, and drawing attention to the systemic and implicit bias present in the fields of both healthcare and education, we have the potential to increase the likelihood that girls' difficulties will be recognised.

## AUTHOR CONTRIBUTIONS

**Jacalyn Guy:** Conceptualization, Formal analysis, Writing – original draft, Writing – review & editing. **Silvana Mareva:** Formal analysis, Writing – review & editing. **Grace Franckel:** Data curation, Writing – review & editing. **the CALM team:** Data curation. **Joni Holmes:** Conceptualization, Methodology, Supervision, Writing – original draft, Writing – review & editing.

## CONFLICT OF INTEREST

The authors have declared that they have no competing or potential conflicts of interest.

## ETHICAL CONSIDERATIONS

Ethical approval was granted by the National Health Service (REC: 13/EE/0157). Parents/caregivers provided written consent and child verbal assent was obtained.

## Supporting information

Supporting Information 1

## Data Availability

Open access to the data from CALM is not yet available as the study is still ongoing. The data will be made available via managed open access once the study is complete. Analysis scripts are available from the corresponding author upon request.

## ENDNOTES

^1^
As defined by the American Psychological Association's Dictionary of Psychology (APA, 2015), sex usually refers to an individual's “biological aspects of maleness or femaleness”, whereas gender connotes the “psychological, behavioural, social, and cultural aspects of being male or female”. We acknowledge that sex can interact with gender in different ways; while some individuals' identities are informed by both sex and gender, others are not (Gur & Gur, 2016; Lai et al., 2015). However, we use the term “sex” throughout the paper, as the majority of neurodevelopmental studies, and indeed our own, record biological sex at birth.

^2^
Identity‐first language is sometimes preferred among people on the autism spectrum (e.g., Kenny et al., 2016). We, therefore, use this terminology throughout the article to respect this preference.
