# Supplementary material for: Dimensions of cognition, behaviour, and mental health in struggling learners: A spotlight on girls
Source: JCPP Adv. 2022 Oct 11;2(4):e12082. doi: 10.1002/jcv2.12082 (PMC10242880; doi:10.1002/jcv2.12082)
Supplement: Supplementary file 1 — Supporting Information 1 [file JCV2-2-e12082-s001.docx]

Table S1

*Measures Used to Derive Dimensions of Cognition, Behaviour, and Mental Health*

|  | Domain | Measure | Reference | Brief Description |
| --- | --- | --- | --- | --- |
| **Cognition** | |  |  |  |
|  | Phonological Processing | PhAB - Alliteration | Frederickson et al., 1997 | A task in which a participant’s ability to isolate initial sounds of simple words is tested. Participants indicate which two of three words began with the same phoneme. |
|  |  | PhAB - Rapid Naming | Frederickson et al., 1997 | A test of speed of phonological production. Participants name pictures of common objects as quickly as possible. |
|  |  | CNRep - Nonword Repetition | Gathercole & Baddeley, 1996 | A test of phonological processing and short-term memory. Participants recall and repeat unfamiliar, non-words. |
|  | WM/STM | AWMA - Digit Recall | Alloway, 2007 | Verbal STM task in which participants repeat strings of numbers of increasing length. |
|  |  | AWMA - Dot Matrix | Alloway, 2007 | Visuo-spatial STM task in which participants recall visual sequences of increasing length. |
|  |  | AWMA - Backward Digit Recall | Alloway, 2007 | Verbal WM tasks in which participants repeat strings of numbers of increasing length in reverse order. |
|  |  | AWMA - Mr X | Alloway, 2007 | Visuo-spatial WM task in which children recall spatial locations of a ball held by a cartoon man rotated in one of seven positions. |
|  |  | Following Instructions | Gathercole et al., 2008 | A span task that requires participants to follow verbal instructions of increasing length. |
|  | Episodic Memory | CMS - Delayed Recall | Cohen, 1997 | A task of episodic memory and language skills in which children listen to a story and retell it in as much detail as possible. |
|  | Processing Speed | TEA-Ch2 - Simple Reaction Time | Manly et al., 2016 | Computerized attention-based reaction time task wherein children pressed a key as soon as a target appeared. |
|  | Executive Function | TEA-Ch2 A/J - Vigil/Barking | Manly et al., 2016 | Computerized sustained attention task wherein participants silently count the number of tones heard throughout a specific trial. |
|  |  | TEA-Ch2 A/J - Cancellation/Balloon Hunt | Manly et al., 2016 | A task of visual selective attention in which participants cross-out target shapes as quickly as possible among distractor shapes. |
|  | Nonverbal Reasoning | WASI-II - Matrix Reasoning | Wechsler, 2011 | A task of fluid reasoning in which participants need to identify the missing part of a puzzle. |
| **Behaviour** | |  |  |  |
|  | Executive Function | BRIEF | Gioia et al., 2000 | Parent ratings of their child’s everyday problem behaviours related to a range of executive function difficulties. |
|  |  | BRIEF - Inhibit |  |  |
|  |  | BRIEF - Shift |  |  |
|  |  | BRIEF - Emotion Regulation |  |  |
|  |  | BRIEF - Initiate |  |  |
|  |  | BRIEF - Working Memory |  |  |
|  |  | BRIEF - Planning |  |  |
|  |  | BRIEF - Organisation |  |  |
|  |  | BRIEF - Monitor^a^ |  |  |
|  | ADHD | CPSF | Conners, 2008 | Parent ratings of ADHD symptomatology in their child. |
|  |  | CPSF - Inattention |  |  |
|  |  | CPSF - Hyperactivity |  |  |
|  |  | CPSF - Learning Problems^b^ |  |  |
|  |  | CPSF - Executive Functions |  |  |
|  |  | CPSF - Aggression |  |  |
|  |  | CPSF - Peer Relations |  |  |
| **Mental Health** | |  |  |  |
|  | Anxiety and Depression | RCADS | Chorpita et al., 2000 | Parent ratings of their child’s symptoms of anxiety and low mood. |
|  |  | RCADS - GAD |  |  |
|  |  | RCADS - Panic Disorder |  |  |
|  |  | RCADS - Social Phobia |  |  |
|  |  | RCADS - Separation Anxiety |  |  |
|  |  | RCADS - OCD |  |  |
|  |  | RCADS - Depression |  |  |
|  | Internalising and Externalising | SDQ | Goodman, 1997 | Parent ratings of their child’s problems relating to externalising and internalising behaviours. |
|  |  | SDQ - Conduct |  |  |
|  |  | SDQ - Prosocial Behaviour |  |  |
|  |  | SDQ - Emotional Symptoms |  |  |
|  |  | SDQ - Peer Relationship Problems |  |  |
|  |  | SDQ - Hyperactivity/Inattention |  |  |

*Note.* All performance-based assessments were classified as cognitive, and all subjective rating-based assessments were classified as behaviour or mental health based on common use in the literature. PhAB = Phonological Assessment Battery; CNRep= Children’s Test of Nonword Repetition; AWMA = Automated Working Memory Assessment; CMS =Children’s Memory Scale; TEA-Ch2 = Test of Everyday Attention for Children 2. BRIEF = The Behaviour Rating Inventory of Executive Function; ADHD = Attention Deficit Hyperactivity Disorder; CPSF = Conners-3 Parent Rating Scale Short Form; RCADS = Revised Child and Anxiety and Depression Scale (Parent Version); SDQ = Strengths and Difficulties Questionnaire; GAD = Generalised Anxiety Disorder; OCD = Obsessive Compulsive Disorder. The Children’s Test of Nonword Repetition (CNRep; Gathercole & Baddeley, 1996) and Revised Child Anxiety and Depression Scale – Parent version (RCADS-P; Chorpita et al., 2000) were introduced in to the study protocol later than all other tasks. The CNRep was introduced after the first 300 participants, and the RCADS-P after the first 390. The Emotion symptoms subscale from the Strengths and Difficulties Questionnaire ( SDQ; Goodman, 1997) was excluded from all analyses, as the items overlapped with those of the RCADS-P (Chorpita et al., 2000). The Learning Problems subscale from the Conners Parent Short Form (CPSF; Conners, 2008) was also dropped because it did not fit easily into one of our three broad domains: it included items such as, “spelling is poor”, tapping into elements of school-based learning that are likely to be influenced by functioning in each of the three broad domains.

Table S2

*Diagnosis and Referral Route*

|  | | | |
| --- | --- | --- | --- |
|  | | | |
| *N* | Boys | Girls | Total |
| **Diagnosis** |  |  |  |
| No diagnosis | 318 | 166 | 484 |
| Diagnosis | 234 | 87 | 321 |
| ADHD | 194 | 61 | 255 |
| ASD | 50 | 7 | 57 |
| Dyslexia | 32 | 15 | 47 |
| **Referrer** |  |  |  |
| Health | 199 | 68 | 267 |
| Education | 332 | 172 | 504 |
| Speech and Language Therapist | 21 | 13 | 34 |
| Total | 552 | 253 | 805 |

*Note.* Some children had multiple diagnoses*.* Referrers in health included paediatricians, child psychiatrists and psychologists, and ADHD nurses. Referrers in education included teachers, special needs coordinators, specialist teachers, educational psychologists. ADHD = Attention Deficit Hyperactivity Disorder; ASD = Autism Spectrum Disorder.

Table S3

*Descriptive Statistics for Whole Sample (N=805) on Cognitive, Behavioural and Mental Health Measures with Raw (left) Standardized (right) Scores*

|  | Raw Scores | | | | | Standardized Scores | | | | |  | |
| --- | --- | --- | --- | --- | --- | --- | --- | --- | --- | --- | --- | --- |
| Measures | *N* | *M* | *SD* | Min | Max | *N* | *M* | *SD* | Min | Max | | Above Cutoff |
|  | | | | | | | | | | | | |
| **Cognition** |  |  |  |  |  |  |  |  |  |  | |  |
| Alliteration | 788 | 7.93 | 2.50 | 0 | 10 | 788 | 91.31 | 10.12 | 69 | 107 | | - |
| Rapid Naming | 786 | 121.53 | 43.03 | 55 | 377 | 787 | 88.68 | 15.13 | 0 | 131 | | - |
| Nonword Repetition | 481 | 25.58 | 6.87 | 2 | 38 | 481 | 82.41 | 20.62 | 45 | 125 | | - |
| Digit Recall | 801 | 24.58 | 5.38 | 7 | 47 | 801 | 92.63 | 15.41 | 60 | 149 | | - |
| Dot Matrix | 799 | 18.03 | 5.69 | 2 | 43 | 799 | 90.63 | 14.98 | 47 | 141.20 | | - |
| Backward Digit Recall | 780 | 9.69 | 4.42 | 0 | 25 | 780 | 91.53 | 12.62 | 58 | 137 | | - |
| MrX | 795 | 9.34 | 5.14 | 0 | 32 | 795 | 97.38 | 15.35 | 61 | 148 | | - |
| Following Instructions | 750 | 11.20 | 4.04 | 1 | 33 | 750 | 0 | 3.64 | -9.60 | 18.58 | | - |
| Delayed Recall | 775 | 28.77 | 16.16 | 0 | 71 | 775 | 7.88 | 3.30 | 1 | 19 | | - |
| Cancellation | 771 | 14.96 | 3.70 | 4.67 | 27 | 771 | 10.17 | 3.31 | 1 | 19 | | - |
| SRT | 739 | 726.54 | 314.23 | 233.51 | 2,276.36 | 739 | 7.80 | 4.06 | 1 | 19 | | - |
| Vigil/Barking | 748 | 7.10 | 2.42 | 0 | 10 | 748 | 8.01 | 3.29 | 3 | 19 | | - |
| Matrix Reasoning | 804 | 11.20 | 5.56 | 0 | 28 | 803 | 43.04 | 9.57 | 20 | 80 | | - |
| **Behaviour** |  |  |  |  |  |  |  |  |  |  | |  |
| BRIEF:Inhibit | 800 | 21.59 | 6.17 | 10 | 30 | 800 | 65.74 | 14.65 | 36 | 98 | | 45.5 |
| BRIEF:Shift | 801 | 17.08 | 4.24 | 8 | 24 | 801 | 68.15 | 14.82 | 36 | 99 | | 50.56 |
| BRIEF:Emotion Control | 800 | 22.18 | 5.57 | 10 | 30 | 800 | 64.70 | 13.35 | 35 | 91 | | 41.88 |
| BRIEF:Initiate | 800 | 17.70 | 3.32 | 8 | 24 | 800 | 66.49 | 10.74 | 35 | 95 | | 40.62 |
| BRIEF:WM | 800 | 25.74 | 4.05 | 11 | 30 | 800 | 73.84 | 9.71 | 38 | 93 | | 72.5 |
| BRIEF:Planning | 789 | 29.02 | 5.00 | 13 | 36 | 789 | 71.40 | 10.46 | 35 | 100 | | 62.74 |
| BRIEF:Organisation | 801 | 14.63 | 3.23 | 6 | 18 | 801 | 60.22 | 10.02 | 33 | 73 | | 17.48 |
| BRIEF:Monitor | 799 | 19.04 | 3.52 | 9 | 24 | 799 | 66.14 | 11.24 | 31 | 91 | | 42.68 |
| CPSF:Inattention | 795 | 11.67 | 3.44 | 0 | 15 | 793 | 80.80 | 11.14 | 40 | 90 | | 83.35 |
| CPSF:Hyperactivity/Impulsivity | 797 | 10.89 | 5.58 | 0 | 18 | 796 | 75 | 15.66 | 40 | 90 | | 64.57 |
| CPSF:Executive Function | 794 | 10.19 | 3.62 | 0 | 15 | 793 | 74.75 | 12.46 | 40 | 90 | | 69.74 |
| CPSF:Aggression | 794 | 3.56 | 3.99 | 0 | 15 | 793 | 64.65 | 17.53 | 44 | 91 | | 37.33 |
| CPSF:Peer Relations | 788 | 5.52 | 4.46 | 0 | 15 | 787 | 73.46 | 17.53 | 44 | 90 | | 60.1 |
| **Mental Health** |  |  |  |  |  |  |  |  |  |  | |  |
| RCADS:Generalised Anxiety | 398 | 5.98 | 3.97 | 0 | 18 | 399 | 56.54 | 12.47 | 36 | 81 | | 19.05 |
| RCADS:Panic Disorder | 396 | 3.91 | 4.12 | 0 | 27 | 397 | 57.44 | 13.64 | 40 | 81 | | 23.93 |
| RCADS:Social Phobia | 400 | 12.44 | 6.34 | 0 | 27 | 401 | 59.55 | 14.18 | 29 | 81 | | 26.18 |
| RCADS:Separation Anxiety | 402 | 6.86 | 5.05 | 0 | 21 | 403 | 59.45 | 14.94 | 36 | 81 | | 28.29 |
| RCADS:Obsessive-Compulsive Disorder | 399 | 3.01 | 3.09 | 0 | 15 | 400 | 53.74 | 11.16 | 41 | 81 | | 11.25 |
| RCADS:Major Depression | 401 | 9.10 | 5.21 | 0 | 27 | 402 | 65.18 | 12.87 | 37 | 81 | | 43.53 |
| SDQ:Conduct Problems | 797 | 3.51 | 2.53 | 0 | 10 |  |  |  |  |  | | 45.09 |
| SDQ:Prosocial Behaviour | 797 | 6.81 | 2.38 | 0 | 10 |  |  |  |  |  | | 41.49 |
| SDQ:Peer Relationships Problem | 797 | 3.43 | 2.62 | 0 | 10 |  |  |  |  |  | | 45.22 |
| SDQ:Hyperactivity/Inattention | 796 | 7.64 | 2.43 | 0 | 10 |  |  |  |  |  | | 60.12 |

*Note.* Descriptive statistics are based on raw scores (left) and standardized scores (right) to ease interpretability. All performance-based assessments were classified as cognitive, and all subjective rating-based assessments were classified as behaviour or mental health based on common use in the literature. For the cognitive tasks, lower raw, standard and scaled scores indicate greater difficulties. However, RAN and SRT are speeded tasks in which higher raw values reflect a slower, and thereby, poorer performance. For all behavioural and mental health tasks, higher raw scores and T-scores reflect greater difficulties. The Prosocial Behaviour Subscale of the SDQ is an exception; higher scores reflect greater strengths. The percentage of participants with scores within the abnormal or clinical levels were calculated using the following criteria: T-scores of 70 or above for all BRIEF, CPSF, RCADS subscales; raw scores equal or higher than 4 for SDQ Conduct problems and Peer relationships problems; raw scores equal or higher than 7 for SDQ Hyperactivity/Inattention; and raw scores equal or lower than 5 for SDQ Prosocial behaviour. Following Instructions is an experimental task that is not age-standardized. To control for age, raw scores were regressed on age and residual scores were used. BRIEF = The Behaviour Rating Inventory of Executive Function; CPSF = Conners-3 Parent Rating Scale Short Form; RCADS = Revised Child and Anxiety and Depression Scale (Parent Version); SDQ = Strengths and Difficulties Questionnaire.

Table S4

*Correlations with Confidence Intervals for all Cognitive Measures (Residual Scores)*

| Variable | 1 | 2 | 3 | 4 | 5 | 6 | 7 | 8 | 9 | 10 | 11 | 12 |
| --- | --- | --- | --- | --- | --- | --- | --- | --- | --- | --- | --- | --- |
| 1. Alliteration |  |  |  |  |  |  |  |  |  |  |  |  |
| 2. Rapid Naming | .29** |  |  |  |  |  |  |  |  |  |  |  |
|  | [.22, .35] |  |  |  |  |  |  |  |  |  |  |  |
| 3. Nonword Repetition | .41** | .24** |  |  |  |  |  |  |  |  |  |  |
|  | [.33, .48] | [.16, .33] |  |  |  |  |  |  |  |  |  |  |
| 4. Digit Recall | .35** | .17** | .53** |  |  |  |  |  |  |  |  |  |
|  | [.29, .41] | [.10, .24] | [.46, .59] |  |  |  |  |  |  |  |  |  |
| 5. Dot Matrix | .25** | .19** | .18** | .32** |  |  |  |  |  |  |  |  |
|  | [.18, .31] | [.12, .26] | [.09, .26] | [.25, .38] |  |  |  |  |  |  |  |  |
| 6. Backward Digit Recall | .39** | .27** | .38** | .46** | .43** |  |  |  |  |  |  |  |
|  | [.33, .45] | [.20, .33] | [.29, .45] | [.41, .52] | [.38, .49] |  |  |  |  |  |  |  |
| 7. MrX | .23** | .15** | .22** | .24** | .40** | .38** |  |  |  |  |  |  |
|  | [.16, .30] | [.08, .22] | [.13, .31] | [.18, .31] | [.33, .45] | [.31, .43] |  |  |  |  |  |  |
| 8. Follow Instructions | .26** | .24** | .33** | .27** | .30** | .31** | .27** |  |  |  |  |  |
|  | [.19, .33] | [.17, .31] | [.24, .41] | [.21, .34] | [.24, .37] | [.24, .38] | [.20, .34] |  |  |  |  |  |
| 9. Delayed Recall | .37** | .18** | .36** | .31** | .24** | .30** | .28** | .28** |  |  |  |  |
|  | [.31, .43] | [.11, .24] | [.28, .44] | [.25, .38] | [.18, .31] | [.23, .36] | [.22, .35] | [.22, .35] |  |  |  |  |
| 10. Cancellation | .23** | .26** | .20** | .15** | .26** | .28** | .20** | .17** | .18** |  |  |  |
|  | [.16, .30] | [.19, .33] | [.11, .28] | [.08, .22] | [.20, .33] | [.21, .34] | [.13, .27] | [.10, .24] | [.11, .25] |  |  |  |
| 11. SRT | .31** | .14** | .19** | .15** | .24** | .25** | .17** | .21** | .21** | .20** |  |  |
|  | [.25, .38] | [.07, .21] | [.10, .28] | [.08, .22] | [.17, .30] | [.18, .31] | [.10, .24] | [.14, .28] | [.14, .28] | [.12, .27] |  |  |
| 12. Vigil | .30** | .14** | .24** | .19** | .24** | .22** | .22** | .20** | .18** | .27** | .23** |  |
|  | [.23, .36] | [.07, .21] | [.15, .33] | [.12, .26] | [.17, .31] | [.15, .29] | [.15, .29] | [.13, .27] | [.11, .25] | [.20, .34] | [.16, .30] |  |
| 13. Matrix Reasoning | .29** | .11** | .27** | .29** | .39** | .37** | .38** | .33** | .34** | .25** | .22** | .24** |
|  | [.23, .36] | [.04, .18] | [.19, .35] | [.23, .35] | [.33, .45] | [.31, .43] | [.32, .43] | [.27, .39] | [.27, .40] | [.18, .31] | [.15, .29] | [.17, .31] |

*Note.* SRT = Simple Reaction Time. Values in square brackets indicate the 95% confidence interval for each correlation. The confidence interval is a plausible range of population correlations that could have caused the sample correlation (Cumming, 2014). * *p* < .05. ** *p* < .01.

Table S5

*Correlations with Confidence Intervals for all Behavioural Measures (Residuals Scores)*

| Variable | 1 | 2 | 3 | 4 | 5 | 6 | 7 | 8 | 9 | 10 | 11 | 12 |
| --- | --- | --- | --- | --- | --- | --- | --- | --- | --- | --- | --- | --- |
| 1. Inhibit |  |  |  |  |  |  |  |  |  |  |  |  |
| 2. Shift | .56** |  |  |  |  |  |  |  |  |  |  |  |
|  | [.51, .60] |  |  |  |  |  |  |  |  |  |  |  |
| 3. Emotion Control | .63** | .71** |  |  |  |  |  |  |  |  |  |  |
|  | [.59, .67] | [.68, .74] |  |  |  |  |  |  |  |  |  |  |
| 4. Initiate | .48** | .58** | .47** |  |  |  |  |  |  |  |  |  |
|  | [.43, .54] | [.53, .63] | [.41, .52] |  |  |  |  |  |  |  |  |  |
| 5. WM | .47** | .47** | .38** | .66** |  |  |  |  |  |  |  |  |
|  | [.41, .52] | [.42, .53] | [.32, .44] | [.62, .70] |  |  |  |  |  |  |  |  |
| 6. Planning | .49** | .49** | .39** | .66** | .72** |  |  |  |  |  |  |  |
|  | [.43, .54] | [.44, .55] | [.33, .45] | [.62, .69] | [.68, .75] |  |  |  |  |  |  |  |
| 7. Organisation | .41** | .33** | .33** | .45** | .49** | .53** |  |  |  |  |  |  |
|  | [.35, .46] | [.27, .39] | [.26, .39] | [.40, .51] | [.43, .54] | [.48, .58] |  |  |  |  |  |  |
| 8. Monitor | .71** | .58** | .58** | .60** | .58** | .69** | .48** |  |  |  |  |  |
|  | [.67, .74] | [.53, .62] | [.53, .62] | [.55, .64] | [.54, .63] | [.65, .72] | [.42, .53] |  |  |  |  |  |
| 9. Inattention | .53** | .39** | .37** | .55** | .72** | .60** | .42** | .61** |  |  |  |  |
|  | [.47, .57] | [.33, .44] | [.30, .43] | [.50, .59] | [.68, .75] | [.56, .65] | [.36, .47] | [.56, .65] |  |  |  |  |
| 10. Hyperactivity/Impulsivity | .78** | .48** | .49** | .42** | .47** | .45** | .39** | .63** | .64** |  |  |  |
|  | [.75, .80] | [.42, .53] | [.44, .54] | [.36, .48] | [.42, .53] | [.40, .51] | [.33, .45] | [.58, .67] | [.60, .68] |  |  |  |
| 11. Executive Function | .46** | .43** | .36** | .60** | .69** | .72** | .64** | .57** | .67** | .51** |  |  |
|  | [.41, .52] | [.37, .48] | [.29, .42] | [.56, .65] | [.65, .73] | [.69, .76] | [.60, .68] | [.52, .61] | [.63, .70] | [.46, .56] |  |  |
| 12. Aggression | .59** | .49** | .61** | .38** | .25** | .30** | .29** | .49** | .31** | .52** | .32** |  |
|  | [.54, .63] | [.44, .54] | [.57, .65] | [.32, .44] | [.19, .32] | [.23, .36] | [.22, .35] | [.43, .54] | [.25, .37] | [.46, .56] | [.26, .38] |  |
| 13. Peer Relations | .46** | .49** | .44** | .49** | .30** | .37** | .23** | .48** | .30** | .37** | .34** | .46** |
|  | [.40, .52] | [.44, .54] | [.39, .50] | [.43, .54] | [.23, .36] | [.31, .43] | [.17, .30] | [.43, .54] | [.23, .36] | [.31, .43] | [.28, .40] | [.40, .51] |

*Note*. WM = working memory. Values in square brackets indicate the 95% confidence interval for each correlation. The confidence interval is a plausible range of population correlations that could have caused the sample correlation (Cumming, 2014).

* *p* < .05. ** *p* < .01.

Table S6

*Correlations with Confidence Intervals for all Mental Health Measures (Residuals Scores)*

| Variable | 1 | 2 | 3 | 4 | 5 | 6 | 7 | 8 | 9 |
| --- | --- | --- | --- | --- | --- | --- | --- | --- | --- |
| 1. Generalised Anxiety Disorder |  |  |  |  |  |  |  |  |  |
| 2. Panic Disorder | .64** |  |  |  |  |  |  |  |  |
|  | [.58, .69] |  |  |  |  |  |  |  |  |
| 3. Social Phobia | .61** | .52** |  |  |  |  |  |  |  |
|  | [.55, .67] | [.44, .59] |  |  |  |  |  |  |  |
| 4. Separation Anxiety | .68** | .59** | .49** |  |  |  |  |  |  |
|  | [.62, .73] | [.52, .65] | [.41, .56] |  |  |  |  |  |  |
| 5. Obsessive-Compulsive Disorder | .65** | .64** | .45** | .53** |  |  |  |  |  |
|  | [.59, .70] | [.57, .69] | [.36, .52] | [.46, .60] |  |  |  |  |  |
| 6. Depression | .64** | .61** | .48** | .64** | .55** |  |  |  |  |
|  | [.58, .70] | [.55, .67] | [.40, .55] | [.57, .69] | [.48, .61] |  |  |  |  |
| 7. Conduct Problems | .30** | .23** | .04 | .26** | .25** | .43** |  |  |  |
|  | [.20, .38] | [.14, .32] | [-.06, .14] | [.17, .35] | [.16, .34] | [.35, .51] |  |  |  |
| 8. Prosocial Behaviour | .17** | .17** | .00 | .26** | .21** | .40** | .52** |  |  |
|  | [.07, .26] | [.07, .26] | [-.10, .10] | [.17, .35] | [.11, .30] | [.31, .48] | [.45, .59] |  |  |
| 9. Peer Relationship Problems | .30** | .30** | .17** | .34** | .33** | .43** | .42** | .41** |  |
|  | [.20, .38] | [.21, .39] | [.07, .26] | [.25, .42] | [.24, .41] | [.34, .50] | [.34, .50] | [.33, .49] |  |
| 10. Hyperactivity/Inattention | .23** | .15** | .03 | .25** | .23** | .39** | .46** | .38** | .28** |
|  | [.13, .32] | [.05, .25] | [-.07, .13] | [.16, .34] | [.13, .32] | [.30, .47] | [.38, .53] | [.29, .46] | [.19, .37] |

*Note.* Values in square brackets indicate the 95% confidence interval for each correlation. The confidence interval is a plausible range of population correlations that could have caused the sample correlation (Cumming, 2014). * indicates *p* < .05. ** indicates *p* < .01.

**Analysis Plan – Additional Information**

Exploratory factor analysis (EFA) with an Oblimin (oblique) rotation was used to identify the number of latent constructs for each set of data (cognitive, behavioural and mental health). The optimal number of factors to retain was chosen based on parallel analysis together with the criterion that each factor required more than one primary loading. Cross loadings were dropped for parsimony and simplicity. Confirmatory factor analysis (CFA) was then used to verify the factor structure. Model fit was evaluated using the chi-squared test (χ2), Comparative Model Fit (CFI), Root Mean Square Error of Approximation (RMSEA) and its confidence interval, and Standardised Root Mean Squared Residuals (SRMR) (see Schermelleh-Engel, Moosbrugger, & Müller, 2003). Modification indices were used to refine model fit. Cross-loadings and covariances were permitted to improve model fit when appropriate. Multigroup CFA was used to determine whether there were differences in model fit between boys and girls. This was achieved using measurement invariance (e.g., van de Schoot et al., 2013).

A series of measurement invariance tests were used to determine whether the cognitive, behavioural, and mental health models derived for the whole sample varied between boys and girls (see Supplementary Table S10). This was achieved by assessing the overall fit of the model with configural invariance, and testing for metric and scalar invariance, which impose equality constraints on the factor loadings and intercepts, respectively. If the constraints of these parameters did not significantly worsen goodness of fit, the models were considered invariant, and the factors were assumed to be measuring the same constructs across boys and girls. In these cases, scalar invariance was met and latent means (or intercepts) were compared between boys and girls to evaluate differences in performance on specific dimensions. If the models were not invariant, modification indices were used to manually release constraints on the models to reach partial scalar invariance, allowing the latent means to be compared between boys and girls (see Figures 1-3).

To compare latent means for boys and girls, latent intercepts in one model were manually constrained in one model and compared to a model in which the intercepts were estimated freely. If the constrained model fit significantly better than the free model, the latent intercepts were assumed equal across groups. Conversely, if the freely estimated model fit better than the constrained model, the latent intercepts were interpreted as different across groups. Individual intercepts were then freed in a stepwise manner to understand specific sex differences in performance. Factor scores were derived from the freely estimated cognitive, behavioural, and mental health models to illustrate similarities and differences in performance.

Table S7

*Factor Loadings for 3- and 4- Factor solutions for Cognitive Measures from Exploratory Factor Analysis*

|  | 4-Factor Solution | | | | 3-Factor Solution | | |
| --- | --- | --- | --- | --- | --- | --- | --- |
|  | Executive | Verbal WM/  STM | Phonological/  Attention | Visual STM | Executive | Speed | Phonological |
|  | | | | | | | |
| Alliteration | 0.20 | 0.12 | **0.44** | -0.16 | 0.04 | **0.59** | 0.09 |
| Rapid Naming | -0.15 | 0.02 | **0.59** | 0.06 | 0.07 | **0.38** | -0.02 |
| Nonword Repetition | 0.13 | **0.48** | 0.21 | -0.27 | -0.12 | **0.40** | **0.50** |
| Digit Recall | -0.02 | **0.87** | -0.04 | 0.04 | 0.10 | -0.04 | **0.80** |
| Dot Matrix | **0.31** | 0.12 | 0.14 | **0.46** | **0.72** | -0.05 | 0.04 |
| Backward Digit Recall | 0.16 | **0.33** | 0.27 | 0.22 | **0.41** | 0.16 | **0.27** |
| Mr X | **0.47** | 0.03 | 0.04 | 0.19 | **0.52** | 0.09 | 0.01 |
| Following Instructions | **0.34** | 0.08 | 0.18 | 0 | **0.26** | **0.27** | 0.08 |
| Delayed Recall | **0.46** | 0.10 | 0.10 | -0.19 | 0.14 | **0.36** | 0.13 |
| Cancellation | 0.09 | -0.08 | **0.44** | 0.13 | **0.26** | **0.34** | -0.12 |
| SRT | 0.22 | -0.09 | **0.33** | -0.01 | 0.18 | **0.40** | -0.13 |
| Vigilance | 0.21 | -0.03 | **0.31** | 0 | 0.18 | **0.38** | -0.07 |
| Matrix Reasoning | **0.68** | 0.01 | -0.05 | 0.05 | **0.47** | 0.18 | 0.04 |
| Eigenvalues | 1.69 | 1.37 | 1.44 | 0.49 | 1.74 | 1.71 | 1.21 |
| % of variance | 13.04 | 10.56 | 11.09 | 3.79 | 13.39 | 13.13 | 9.31 |

*Note.* SRT = Simple Reaction Time. Loadings above 0.25 are presented in bold.

Table S8

*Factor Loadings for 3- Factor solution for Behavioural Measures (with and without Monitor Subscale) from Exploratory Factor Analysis*

|  | 3-Factor Solution with Monitor Subscale | | | 3-Factor Solution without Monitor Subscale | | |
| --- | --- | --- | --- | --- | --- | --- |
| Measures | Cognitive Control | Emotional Regulation | Behavioural Regulation | Cognitive Control | Emotional Regulation | Behavioural Regulation |
| Inhibition | 0.06 | **0.39** | **0.58** | 0.06 | **0.36** | **0.60** |
| Shift | 0.20 | **0.71** | -0.03 | 0.23 | **0.69** | -0.02 |
| Emotion Regulation | -0.01 | **0.80** | 0.10 | 0 | **0.82** | 0.08 |
| Initiation | **0.66** | **0.33** | -0.14 | **0.68** | **0.29** | -0.12 |
| Working Memory | **0.86** | -0.02 | 0.01 | **0.86** | -0.01 | 0 |
| Planning | **0.84** | 0.09 | -0.06 | **0.86** | 0.07 | -0.06 |
| Organisation | **0.56** | 0.04 | 0.06 | **0.57** | 0.04 | 0.06 |
| Monitor | **0.43** | **0.34** | 0.24 | - | - | - |
| Inattention | **0.66** | -0.15 | **0.37** | **0.66** | -0.13 | **0.36** |
| Hyperactivity/Impulsivity | 0.09 | 0.07 | **0.84** | 0.08 | 0.07 | **0.88** |
| Executive Functions | **0.82** | -0.06 | 0.08 | **0.82** | -0.05 | 0.08 |
| Aggression | -0.11 | **0.59** | **0.29** | -0.1 | **0.61** | **0.27** |
| Peer Relations | 0.14 | **0.51** | 0.03 | 0.14 | **0.51** | 0.03 |
| Eigenvalues | 4.00 | 2.65 | 1.82 | 3.68 | 2.47 | 1.65 |
| % of variance | 30.79 | 20.39 | 13.98 | 30.68 | 20.62 | 13.79 |

*Note.* Loadings above 0.25 are presented in bold.

Table S9

*Factor Loadings for 2- Factor solution for Mental Health Measures from Exploratory Factor Analysis*

| Measures | Internalising | Externalising |
| --- | --- | --- |
| Generalised Anxiety Disorder | **0.86** | 0 |
| Panic Disorder | **0.78** | 0 |
| Social Phobia | **0.78** | -0.25 |
| Separation Anxiety | **0.73** | 0.11 |
| Obsessive-Compulsive Disorder | **0.71** | 0.07 |
| Depression | **0.64** | **0.34** |
| Conduct Problems | 0.03 | **0.73** |
| Prosocial Behaviour | -0.05 | **0.73** |
| Peer Relationship Problems | 0.20 | **0.48** |
| Hyperactivity/Inattention | 0.04 | **0.56** |
| Eigenvalues | 3.53 | 1.90 |
| % of variance | 35.35 | 19.04 |

*Note.* Loadings above 0.25 are presented in bold.

Table S10

*Fit statistics for Measurement Invariance of Cognitive, Behavioural and Mental Health Models in Boys and Girls*

|  | Model | n | χ^2^ | df | *p* | Δχ2 | Δdf | *p* | CFI | RMSEA  (90% CI) | AIC |
| --- | --- | --- | --- | --- | --- | --- | --- | --- | --- | --- | --- |
| Cognitive | |  |  |  |  |  |  |  |  |  |  |
|  | Configural Invariance | 805 | 280.7 | 124 | <.001 | - | - | - | 0.922 | 0.056  [ 0.047 0.065] | 64050 |
|  | Metric Invariance | 805 | 293.16 | 134 | <.001 | 11.48 | 10 | .321 | 0.921 | 0.054  [0.046 0.063] | 64043 |
|  | Scalar Invariance | 805 | 311.6 | 144 | <.001 | 18.86 | 10 | .042 | 0.916 | 0.054  [0.046 0.062] | 64041 |
|  | Partial Scalar Invariance^a^ | 805 | 300.88 | 143 | <.001 | 7.85 | 9 | .550 | 0.921 | 0.052  [0.044 0.061] | 64032 |
| Behavioural | |  |  |  |  |  |  |  |  |  |  |
|  | Configural Invariance | 805 | 530.82 | 98 | <.001 | - | - | - | 0.930 | 0.105  [0.096 0.114] | 49099 |
|  | Metric Invariance | 805 | 546.52 | 108 | <.001 | 15.79 | 10 | .106 | 0.929 | 0.101  [0.092 0.109] | 49095 |
|  | Scalar Invariance | 805 | 578.80 | 117 | <.001 | 31.68 | 9 | .0002 | 0.925 | 0.099  [0.091 0.107] | 49109 |
|  | Partial Scalar Invariance^b^ | 805 | 558.52 | 114 | <.001 | 11.69 | 6 | .07 | 0.928 | 0.099  [0.090 0.107] | 49095 |
| Mental Health | |  |  |  |  |  |  |  |  |  |  |
|  | Configural Invariance | 403 | 147.69 | 66 | <.001 | - | - | - | 0.954 | 0.078  [0.061 0.095] | 19731 |
|  | Metric Invariance | 403 | 156.06 | 75 | <.001 | 6.75 | 9 | .663 | 0.955 | 0.073  [0.057 0.089] | 19721 |
|  | Scalar Invariance | 403 | 171.62 | 83 | <.001 | 15.20 | 8 | .06 | 0.951 | 0.073  [0.057 0.088] | 19721 |

*Note.* ^a^ Model includes free estimation of intercepts for Mr X task. ^b^ Model includes free estimation of intercepts for Organisation, Planning and Working Memory subscales.

**References**

Alloway, T., Gathercole, S. E., & Pickering, S. J. (2006). Verbal and visuospatial short‐term and working memory in children: Are they separable? *Child Development*, *77*(6), 1698–1716. <https://doi.org/https://doi.org/10.1111/j.1467-8624.2006.00968.x>

Chorpita, B. F., Yim, L., Moffitt, C., Umemoto, L. A., & Francis, S. E. (2000). Assessment of symptoms of DSM-IV anxiety and depression in children: a revised child anxiety and depression scale. *Behaviour Research and Therapy*, *38*(8), 835–855. https://doi.org/10.1016/S0005-7967(99)00130-8

Cohen, M. (1997). *Children’s memory scale (CMS)*. The Psychological Corporation.

Conners, C. (2008). Conners 3rd edition (Conners 3). *Toronto, ON: Multi-Health Systems*.

Frederickson, N., Frith, U., & Reason, R. (1997). *Phonological Assessment Battery (PhAB): Manual and Test Materials*. NFERNelson.

Gathercole, S. E., & Baddeley, A. D. (1996). *The children’s test of nonword repetition*. Pearson.

Gathercole, S. E., Durling, E., Evans, M., Jeffcock, S., & Stone, S. (2008). Working memory abilities and children’s performance in laboratory analogues of classroom activities. *Applied Cognitive Psychology: The Official Journal of the Society for Applied Research in Memory and Cognition*, *22*(8), 1019–1037. <https://doi.org/10.1002/acp.1407>

Gioia, G. A., Isquith, P. K., Guy, S. C., & Kenworthy, L. (2000). *Behavior rating inventory of executive function: BRIEF*. Psychological Assessment Resources Odessa, FL.

Goodman, R. (1997). The Strengths and Difficulties Questionnaire: A research note. *Journal of Child Psychology and Psychiatry*, *38*(5), 581–586. https://doi.org/https://doi.org/10.1111/j.1469-7610.1997.tb01545.x

Manly, T., Anderson, V., Crawford, J., George, M., Underbjerg, M., & Robertson, I. (2016). Test of everyday attention for children. (TEA–Ch2) London. *UK: Harcourt Assessment*.

van de Schoot, R., Kluytmans, A., Tummers, L., Lugtig, P., Hox, J., & Muthén, B. (2013). Facing off with Scylla and Charybdis: a comparison of scalar, partial, and the novel possibility of approximate measurement invariance. *Frontiers in Psychology*, *4*. https://doi.org/10.3389/fpsyg.2013.00770

Wechsler, D. (2011). *WASI-II: Wechsler abbreviated scale of intelligence*. PsychCorp.
